# Healthy eating index-2015 and its association with the prevalence of stroke among US adults

**DOI:** 10.1038/s41598-024-54087-9

**Published:** 2024-02-12

**Authors:** Xiao-Fei Wu, Fei Yin, Gui-Jie Wang, Ye Lu, Rong-Fei Jin, Dong-Lin Jin

**Affiliations:** Department of Emergency Care Medicine, Suzhou Ninth People’s Hospital, Suzhou, 215299 Jiangsu China

**Keywords:** Stroke, Healthy eating index, NHANES, Dietary patterns, LASSO, Genetics, Neuroscience, Cardiology, Diseases, Health care, Medical research, Risk factors

## Abstract

This study aims to investigate the relationship between the healthy eating index (HEI) and the prevalence of stroke within a diverse United States population. Employing a cross-sectional design, we utilized data sourced from the National Health and Nutrition Examination Survey (NHANES). Dietary information was collected from participants and HEI scores were computed. NHANES employed stratified multistage probability sampling, with subsequent weighted analysis following NHANES analytical guidelines. Thorough comparisons were made regarding the baseline characteristics of individuals with and without stroke. Weighted multivariable logistic regression analysis and restricted cubic spline (RCS) methods were employed to ascertain the association between stroke risk and HEI, with LASSO regression utilized to identify dietary factors most closely linked to stroke risk. Additionally, we constructed a nomogram model incorporating key dietary factors and assessed its discriminatory capability using the receiver operating characteristic (ROC) curve. Our study encompassed 43,978 participants, representing an estimated 201 million U.S. residents. Participants with a history of stroke exhibited lower HEI scores than their non-stroke counterparts. Logistic regression analysis demonstrated a robust association between lower HEI scores and stroke, even after adjusting for confounding variables. RCS analysis indicated a nonlinear negative correlation between HEI and stroke risk. Furthermore, detailed subgroup analysis revealed a significant gender-based disparity in the impact of dietary quality on stroke risk, with females potentially benefiting more from dietary quality improvements. Sensitivity analysis using unweighted logistic regression yielded results consistent with our primary analysis. The nomogram model, based on key dietary factors identified through LASSO regression, demonstrated favorable discriminatory power, with an area under the curve (AUC) of 79.3% (95% CI 78.4–81.2%). Our findings suggest that higher HEI scores are inversely related to the risk of stroke, with potential greater benefits for women through dietary quality enhancement. These results underscore the importance of improving dietary quality for enhanced stroke prevention and treatment.

## Introduction

Stroke is a devastating medical condition and one of the leading causes of death and disability worldwide. According to the World Health Organization (WHO), stroke affects approximately 15 million people each year, resulting in 5 million deaths and 5 million people being permanently disabled^1^. In addition, stroke places a significant economic burden on society, with estimates of the total cost of stroke ranging from $28 billion to $68 billion annually in the United States alone^2^. Therefore, identifying modifiable risk factors for stroke is of great public health importance. Dietary habits are among the modifiable risk factors that have been extensively studied for their role in the prevention of stroke^3–5^. A healthy diet, characterized by a high intake of fruits, vegetables, whole grains, and lean protein sources, has been shown to be associated with a reduced risk of stroke^6,7^. Several dietary indices have been developed to assess overall diet quality, such as the Dietary Approaches to Stop Hypertension (DASH) diet, the Mediterranean diet, and the Healthy Eating Index (HEI)^7–9^. Moreover, there are gender differences in the impact of dietary quality on the occurrence of various diseases. Several studies have shown that women are more susceptible to the negative effects of a poor diet, particularly in the context of cardiovascular disease (CVD)^10,11^.

The HEI is a measure of diet quality that assesses adherence to the United States Department of Agriculture’s (USDA) dietary guidelines^5,12^. The HEI is based on 13 components, including the intake of fruits, vegetables, whole grains, dairy, protein foods, seafood, and added sugars, as well as the ratio of unsaturated to saturated fats and sodium intake^13,14^. The HEI has been widely used in epidemiological studies to evaluate the association between diet quality and chronic disease outcomes, including cardiovascular disease, diabetes, and cancer^14–16^. Although the association between diet quality and the risk of stroke has been investigated in several studies, the results have been inconsistent. Some studies have found that a better dietary quality is associated with a lower risk of stroke. Moreover, most of the previous studies have been conducted without a standard measurement to evaluate the dietary quality, and few study on the association between HEI and stroke^17,18^.

Therefore, the aim of this study was to investigate the association between HEI scores and the risk of stroke in a population-based sample of adults in the United States. We hypothesized that a higher HEI score would be associated with a lower risk of stroke. The findings of this study could provide insights into the potential role of diet quality in stroke prevention and inform the development of public health strategies to improve diet quality and reduce the burden of stroke.

## Methods

### Study population

NHANES, a continuous cross-sectional survey, conducted by the National Center for Health Statistics in the Centers for Disease Control and Prevention once every 2 years to assess the health and nutrition status of residents in the U.S. NHANES team employed “stratified multistage probability sampling” method to ensure participants enrolled were representative. The detailed method description can be found on the NHANES official website (http://www.cdc.gov/nchs/nhanes.htm, last accessed on 18 Mar 2023)^19^. 10 consecutive study circles of NHANES from 1999/2000 to 2017/2018 were included in the present study. The exclusion criteria were as follows: (1) age < 18 or ≥ 80 years, (2) participants without stroke status, (3) pregnant individuals, (4) participants without dietary data or missing.

### Dietary information

The dietary interview, referred to as What We Eat in America (WWEIA), is conducted collaboratively by the US Department of Agriculture (USDA) and the US Department of Health and Human Services (DHHS). All eligible NHANES participants undergo two 24-h dietary recall interviews to disclose the types and amounts of foods they consumed in the 24 h prior to the interview (from midnight to midnight). These interviews aim to gather information about the quantities and varieties of foods individuals ingested in the 24 h leading up to the interview, covering the time span from midnight to midnight. The first dietary recall is conducted face-to-face at the Mobile Examination Center (MEC), while the second recall occurs through a telephone interview approximately 3 to 10 days after the initial recall. In this study, participants’ daily intake of HEI components is determined by averaging their two dietary recalls. The USDA’s Food and Nutrient Database for Dietary Studies (FNDDS) is employed to compute the nutrients and food components in all food items. The dataset containing total nutrient intakes serves as a concise record of the nutrient intake for each individual. The NHANES data from 1999 to 2018 was collected to gather information on the participants’ dietary intake, including food frequency questionnaires, 24-h dietary recall, and supplement intake. The HEI version 2015 was employed for assessing dietary quality. HEI ranges from 0 to 100, the higher HEI scores, the better quality of diet. The HEI score is calculated based on ten components, which are grouped into two categories: Adequacy and Moderation. Adequacy components are further divided into four subcategories: total fruit, whole fruit, total vegetables, and greens and beans. Moderation components are divided into five subcategories: whole grains, dairy, total protein foods, fatty acids, and added sugars. Each component has a specific set of standards and guidelines, and the score for each component ranges from 0 to 5, depending on how well the participant meets the recommendations. The component scores are summed to create a total score ranging from 0 to 100. The HEI score is then used to analyze the association between the quality of the diet and the risk of stroke, by comparing the scores of participants who have had a stroke with those who have not. Due to the wide range of HEI-2015, which spans from 0 to 100, the increase in the prevalence of stroke for each incremental unit of HEI-2015 is minimal. The decision to categorize HEI scores into quartiles was based on the desire to explore potential associations between dietary quality, as assessed by the HEI, and the risk of stroke across different levels of dietary quality. We have calculated and present the quartiles of HEI scores for both the total sample and stratified by stroke and non-stroke groups. The quartile boundaries for the total sample, stroke, and non-stroke groups are as follows: Total Sample: Q1: (0–26.1]; Q2: (26.1–51.3]; Q3: (51.3–72.6]; Q4: (72.6–96.7]. Stroke Group: Q1: (0–23.2]; Q2: (23.2–47.8]; Q3: (47.8–70.3]; Q4: (70.3–93.2]. Non-Stroke Group: Q1: (0–28.8], Q2: (28.8–53.2], Q3: (53.2–76.9]; Q4: (76.9–96.7].

### Definition of stroke

Like previous articles published using NHANES data, the primary outcome of this study was determined based on self-reported history of stroke. Participants were asked in the health questionnaire whether a healthcare professional had ever informed them of a previous stroke diagnosis. Those who responded affirmatively were classified as having had a stroke.

### Covariates

Structured questionnaires and face-to-face interviews were employed to collect demographic information, encompassing age, gender, racial/ethnic background, educational level, physical inactivity, smoking status, and patterns of alcohol consumption. Participants meeting any of the following criteria were categorized as having hypertension: (1) Mean systolic blood pressure (SBP) ≥ 140 mmHg; (2) Mean diastolic blood pressure (DBP) ≥ 90 mmHg; (3) Self-reported diagnosis of hypertension; (4) Current use of antihypertensive medications. Additionally, diabetes served as a notable confounding factor with potential implications for stroke. Those with a prior diagnosis of diabetes by a physician or health professional were classified as having diagnosed diabetes. Individuals without a history of diagnosed diabetes but demonstrating HbA1c levels of 6.5% (47.5 mmol/mol) or higher, fasting plasma glucose (FPG) levels of 126 mg/dL (7.0 mmol/L) or higher, or 2-h oral glucose tolerance test (OGTT) plasma glucose levels of 200 mg/dL or higher (11.1 mmol/L), as determined by laboratory tests, were also designated as having diabetes. All blood samples were collected after at least an 8-h overnight fast to examine blood biochemical indexes. Dyslipidemia was defined as the presence of one of the conditions below in the participants: (1) self-reported dyslipidemia, (2) currently using hypolipidemic drugs, (3) total cholesterol (TC) ≥ 200 mg/dL, (4) TG ≥ 150 mg/dL, (5) LDL-C ≥ 130 mg/dL, and (6) HDL-C < 50 mg/dL (female) or < 40 mg/dL (male). According to WHO guidelines, normal weight, overweight, and obesity were defined as participants with a BMI < 25 kg/m^2^, 25 ≤ BMI < 30 kg/m^2^, and BMI ≥ 30 kg/m^2^, respectively. We used the Chronic Kidney Disease Epidemiology Collaboration creatinine equation to calculate the estimated glomerular filtration rate (eGFR).

### Statistical analysis

NHANES analytic and reporting guidance was followed for all analysis procedures. Stratified multistage probability sampling was utilized in NHANES to reduce bias caused by post-stratification, non-response, and oversampling. To ultimately produce representative estimates nationwide, each participant was assigned a specific sampling weight based on the primary sampling unit. In this study, all analysis was produced according to the final 20-year survey weight in statistical analysis. Weighted mean (95% confidence interval (CI)) was used to present continuous variables, while proportions (95% CI) were used to represent categorical variables. Adjusted Wald test for continuous variables and Rao–Scott χ^2^ test for categorical variables were used to compare baseline characteristics. HEI components were compared using the adjusted Wald test. Considering the potential inflation of Type I error due to the numerous hypothesis tests performed, it is crucial to employ a correction method to mitigate this concern. The Bonferroni correction was used. Weighted multivariable logistic regression analysis was adopted to assess the association of HEI with stroke. In order to assess the non-linear relationships between HEI and the risk of stroke, a restricted cubic spline (RCS) analysis was conducted with three piecewise points, using the median value of each anthropometric measurement as the reference point. Subgroup analysis was conducted to evaluate heterogeneity, stratified by age, sex, BMI, and race. LASSO regression was chosen for its ability to handle variable selection, address multicollinearity, and improve the overall robustness and interpretability of the regression model in the context of our study. LASSO regression based on the “*glmnet*” package in R was carried out, and the nomogram model was established based on the “*replot*” package. All dietary components that contributed to HEI were included in the LASSO regression model, along with three important demographic variables (age, sex, and race). The penalty parameter lambda (λ) was selected based on tenfold cross-validation, with 1000 iterations performed to ensure accuracy. As in previous studies, the largest λ within one standard error range of the minimal binomial deviation (λ = 0.0005925861) was chosen as the optimal model. The discriminatory power of the nomogram model in identifying hypertension risk was evaluated with the receiver operating characteristic (ROC) curve. “Multiple imputation” was adopted to fill in missing covariates and to avoid selection bias due to excluding participants with missing data. We utilized the “mice” R package for multiple imputation in this study, where all missing data were completed at random. For each variable with missing data, it was treated as the dependent variable, while the other variables were considered independent variables, and imputation was performed individually for each variable. Multiple imputations were conducted, resulting in the generation of five complete datasets to better capture the uncertainty introduced by the missing data. Subsequently, by computing the mean or median and considering the weights assigned to each dataset, we aggregated the results from the multiple imputed datasets. A P value < 0.05 was considered significant, and R software (version 4.1.6, R Foundation for Statistical Computing, Vienna, Austria) was used for all statistical analyses.

### Ethics approval and consent to participate

The NCHS Ethics Review Board protects the rights and welfare of NHANES participants. The NHANES protocol complies with the U.S. Department of Health and Human Services Policy for the Protection of Human Research Subjects. NCHS IRB/ERC Protocol number: 2011-17. Ethical review and approval were waived for this study as it solely used publicly available data for research and publication.

## Results

### Characteristics of the study population

**Figure 1:**
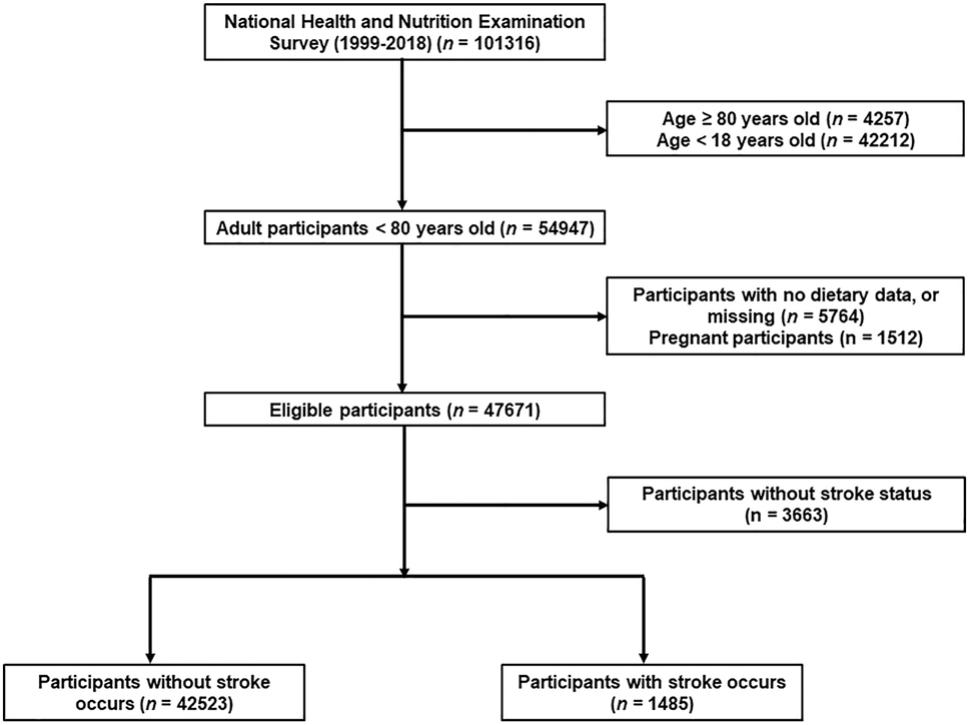
Flowchart of the study population.

In the current study, 43,978 individuals from NHANES (1999–2018) were included, representing 201 million (survey samples were 201,162,632) individuals with multiple racial ancestries in the United States (Fig. 1). Of these individuals, 1485 once had a stroke. Among all participants, 49.31% were male, 68.27% were non-Hispanic white, and the mean age was 45.82 years. Significant differences in demographic and clinical characteristics were observed between participants with and without stroke. Participants with stroke were significantly older (60.27 vs. 45.46 years old, P < 0.001), had lower education levels, and a higher proportion of smokers. The prevalence of diabetes was also higher in participants with stroke than in those without stroke (35.74% vs. 11.74%, P < 0.001). In addition, the eGFR was significantly lower in participants with stroke compared to those without stroke (77.37 vs. 95.67 mL/min/1.73 m^2^, P < 0.001). The detailed characteristics of the study population grouped by stroke status and HEI quartiles are presented in Table 1 and Table S1. We calculated HEI and found that participants in the stroke group had a lower mean HEI than those without stroke (48.90 vs. 50.21, P = 0.004); the standardized mean difference was 0.105. In addition, we also compared important cardiovascular and cerebrovascular biomarkers among participants in different quartiles of HEI scores. We found that participants with higher HEI had cardiovascular and metabolic conditions compared with participants with lower HEI scores (Table S2). To explore further the dietary factors that contributed to the difference in HEI between the two groups, we compared each component score of HEI, as shown in Table 2. Individuals with stroke had a lower HEI score in greens and beans, seafood and plant proteins, fatty acids, and saturated fatty acids.

**Table 1:** Baseline characteristics of 43,978 individuals enrolled in the cross-sectional study.

Variables	Overall (n = 43,978)	Non-stroke (n = 42,523)	Stroke (n = 1485)	*P* value
Age, years	45.82 [45.49, 46.16]	45.46 [45.12, 45.80]	60.27 [59.39, 61.14]	< 0.001***
Sex-male, *n* (%)	49.31 [47.59, 51.03]	49.41 [48.93, 49.89]	45.35 [42.23, 48.48]	0.01*
Race, *n* (%)				< 0.001***
Non-Hispanic White	68.27 [64.22, 72.32]	68.31 [66.29, 70.33]	66.72 [63.08, 70.36]	
Non-Hispanic Black	11.18 [10.24, 12.13]	11.04 [9.94, 12.14]	16.80 [14.49, 19.12]	
Mexican American	8.21 [7.24, 9.17]	8.28 [7.22, 9.34]	5.28 [3.96, 6.59]	
Other Hispanic	5.67 [4.82, 6.53]	5.72 [4.85, 6.60]	3.65 [2.37, 4.93]	
Other	6.66 [6.12, 7.21]	6.64 [6.07, 7.21]	7.54 [5.51, 9.58]	
Smoking, *n* (%)	22.27 [21.18, 23.35]	22.09 [21.33, 22.85]	29.66 [26.61, 32.71]	< 0.001***
Drinking, *n* (%)	83.43 [80.33, 86.53]	89.31 [88.47, 90.15]	84.49 [81.76, 87.23]	< 0.001***
Education level, *n* (%)				< 0.001***
Below high school	5.41 [5.01, 5.80]	5.29 [4.89, 5.69]	10.30 [8.30, 12.29]	
High school	35.23 [33.49, 36.98]	34.94 [33.76, 36.13]	47.68 [44.36, 51.00]	
Above high school	59.29 [56.86, 61.72]	59.77 [58.43, 61.11]	42.02 [38.51, 45.53]	
SBP, mmHg	121.61 [121.30, 121.92]	121.39 [121.08, 121.71]	130.20 [128.69, 131.71]	< 0.001***
DBP, mmHg	71.65 [71.36, 71.94]	71.66 [71.38, 71.95]	71.02 [70.14, 71.91]	0.15
Diabetes, *n* (%)	12.33 [11.75, 12.92]	11.74 [11.29, 12.20]	35.74 [32.50, 38.98]	< 0.001***
FBG, mmol/L	5.84 [5.80, 5.87]	5.81 [5.78, 5.85]	6.63 [6.40, 6.86]	< 0.001***
HbA1c, %	5.57 [5.55, 5.58]	5.56 [5.54, 5.57]	6.07 [5.98, 6.16]	< 0.001***
eGFR, mL/min/1.73 m^2^	95.23 [94.74, 95.71]	95.67 [95.18, 96.16]	77.37 [75.69, 79.04]	< 0.001***
TG, mmol/L	1.50 [1.47, 1.53]	1.49 [1.47, 1.52]	1.73 [1.60, 1.85]	< 0.001***
TC, mmol/L	5.08 [5.06, 5.10]	5.09 [5.07, 5.10]	4.97 [4.89, 5.05]	0.01*
LDL-C, mmol/L	3.01 [2.99, 3.03]	3.01 [2.99, 3.03]	2.86 [2.76, 2.96]	0.003**
HDL-C, mmol/L	1.37 [1.36, 1.37]	1.37 [1.36, 1.38]	1.32 [1.28, 1.35]	0.001**
RBC, × 10^9^/L	4.73 [4.72, 4.74]	4.73 [4.72, 4.74]	4.59 [4.55, 4.64]	< 0.001***
WBC, × 10^9^/L	7.26 [7.21, 7.30]	7.25 [7.20, 7.29]	7.52 [7.39, 7.66]	< 0.001***
NE, × 10^9^/L	4.30 [4.27, 4.33]	4.29 [4.26, 4.32]	4.59 [4.48, 4.70]	< 0.001***
Monocyte, × 10^9^/L	0.56 [0.56, 0.56]	0.56 [0.56, 0.56]	0.59 [0.58, 0.61]	< 0.001***
LY, × 10^9^/L	2.15 [2.13, 2.16]	2.15 [2.14, 2.17]	2.07 [2.02, 2.12]	0.001**
PLT, × 10^6^/L	254.86 [253.51, 256.22]	255.02 [253.68, 256.37]	248.55 [242.37, 254.74]	0.04*
Hemoglobin, g/L	14.37 [14.33, 14.41]	14.38 [14.34, 14.42]	14.01 [13.89, 14.13]	< 0.001***

Continuous variables are presented as the mean [95% CI], category variables are presented as the proportion [95% CI].

*CI* confidence interval, *SBP* systolic blood pressure, *DBP* diastolic blood pressure, *FBG* fasting blood glucose, *HbA1c* glycated hemoglobin, *eGFR* estimated glomerular filtration rate, *TG* triglycerides, *TC* total cholesterol, *LDL-C* low-density lipoprotein cholesterol, *HDL-C* high-density lipoprotein cholesterol, *RBC* red blood cells, *WBC* white blood cells, *NE* neutrophils, *LY* lymphocytes, *PLT* platelets.

*P value < 0.05, **P value < 0.01, ***P value < 0.001.

**Table 2:** Comparison of each component of HEI scores between stroke and none-stroke group.

Variables	Overall (n = 43,978)	Non-stroke (n = 42,523)	Stroke (n = 1485)	*P* value
HEI	50.18 [49.85, 50.51]	50.21 [49.88, 50.55]	48.90 [48.01, 49.79]	0.004**
Total vegetables	3.05 [3.02, 3.07]	3.05 [3.02, 3.08]	2.96 [2.85, 3.07]	0.14
Greens and beans	1.46 [1.42, 1.50]	1.46 [1.42, 1.50]	1.20 [1.06, 1.34]	< 0.001***
Total fruits	2.03 [1.99, 2.08]	2.03 [1.99, 2.07]	2.10 [1.97, 2.23]	0.31
Whole fruits	2.08 [2.04, 2.13]	2.09 [2.04, 2.13]	2.07 [1.92, 2.21]	0.81
Whole grains	2.23 [2.18, 2.29]	2.23 [2.17, 2.29]	2.41 [2.15, 2.67]	0.18
Dairy	4.94 [4.88, 5.00]	4.94 [4.88, 5.00]	4.73 [4.48, 4.97]	0.1
Total protein foods	4.18 [4.16, 4.20]	4.18 [4.16, 4.20]	4.11 [4.02, 4.20]	0.15
Seafood and plant proteins	2.25 [2.21, 2.28]	2.25 [2.21, 2.29]	2.05 [1.91, 2.19]	0.01*
Fatty acids	4.96 [4.90, 5.02]	4.97 [4.91, 5.03]	4.68 [4.43, 4.92]	0.02*
Sodium	4.59 [4.54, 4.64]	4.59 [4.54, 4.65]	4.47 [4.19, 4.75]	0.4
Refined grains	6.07 [6.01, 6.13]	6.07 [6.01, 6.13]	6.29 [6.02, 6.56]	0.11
Saturated fatty acids	5.94 [5.88, 6.00]	5.95 [5.89, 6.01]	5.54 [5.30, 5.78]	0.001**
Added sugars	6.40 [6.32, 6.48]	6.41 [6.33, 6.48]	6.31 [6.06, 6.55]	0.43

Data are presented as the mean [95% CI].

*CI* confidence interval, *HEI* healthy eating index.

***P value < 0.001, **P value < 0.01, *P value < 0.05.

### Association of HEI with risk of stroke

**Figure 2:**
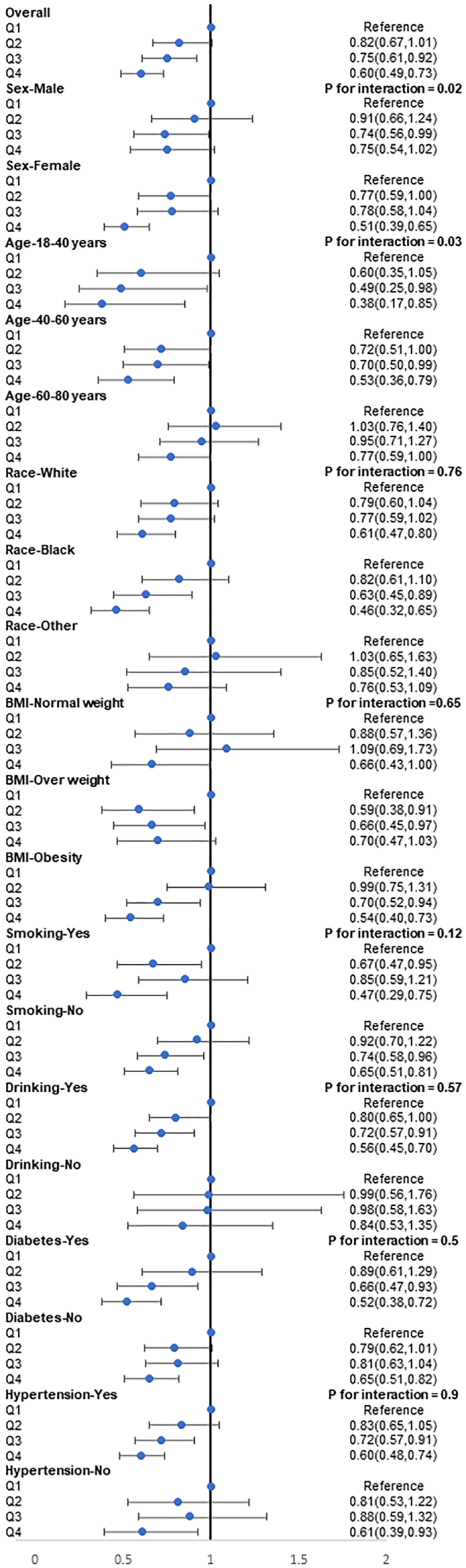
Subgroup analyses for the association of HEI with the risk of stroke. Multivariable weight Logistic analyses were conducted among different populations after adjusting for age, sex, race/ethnicity, smoking, drinking, education levels, hypertension, diabetes, dyslipidemia, physical inactivity, and obesity. *HEI* health eating index.

**Figure 3:**
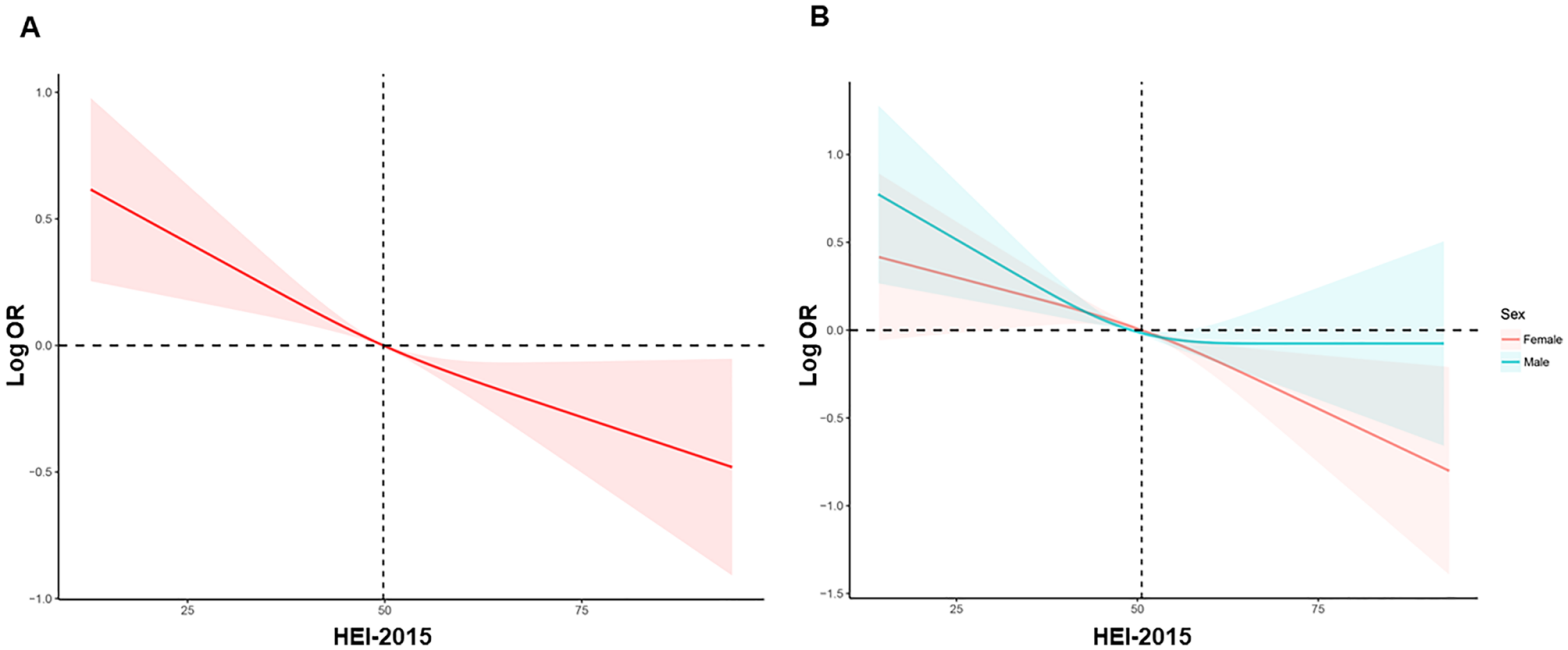
RCS analysis on the association between HEI and the risk of stroke. (**A**) RCS curve of the association between HEI and stroke among all participants; (**B**) subgroup RCS analysis stratified by sex. *RCS* restricted cubic spline, *HEI* health eating index.

Weighted logistic regression was utilized in the current study to examine the correlation between HEI and stroke. The participants were categorized into quartiles based on their HEI levels, and the logistic regression results showed that individuals with higher HEI levels had a decreased risk of developing stroke compared to those with lower HEI levels, before and after adjusting for confounding factors, including age, sex, ethnicity, smoking, drinking, education levels, hypertension, and diabetes (Table 3). The results indicated that higher HEI was associated with a decreased risk of stroke. Furthermore, the study conducted detailed subgroup analyses by stratifying the data by sex, age (18–40, 40–60, 60–80 years old), race, BMI (normal weight, overweight, obesity), smoking status, drinking status, diabetes, and hypertension to explore the association between HEI and stroke among different populations. The results indicated that there were no significant differences in the association of HEI and stroke across different race groups, weight groups, smoking groups, drinking groups, diabetes groups, and hypertension groups, suggesting that the study's conclusion was consistent. However, we found that compared to male individuals, a high healthy diet index may benefit female individuals more (*P* for interaction = 0.02) (Fig. 2). Results of RCS analysis also demonstrated that HEI was negatively correlated with the risk of stroke, and in a nonlinear pattern. The risk of stroke decreased rapidly with the increase of HEI; however, the decrease in stroke risk for men after the median age is not significant compared with women (Fig. 3). The above results suggest that the impact of dietary quality on stroke incidence may be greater for women than for men.

**Table 3:** Logistic regression analysis on the association between HEI and stroke.

	Non-adjusted model		Model I		Model II	
	OR [95% CI]	*P* value	OR [95% CI]	*P* value	OR [95% CI]	*P* value
Q1	Reference	–	Reference	–	Reference	–
Q2	0.91 (0.75, 1.11)	0.35	0.77 (0.62, 0.94)	0.01	0.86 (0.62, 1.08)	0.07
Q3	0.86 (0.70, 1.04)	0.13	0.63 (0.51, 0.77)	< 0.001***	0.77 (0.61, 0.96)	0.01**
Q4	0.76 (0.63, 0.92)	0.005**	0.47 (0.39, 0.56)	< 0.001***	0.65 (0.52, 0.76)	< 0.001***

Data are presented as OR [95% CI]. Model I adjusted for age, sex, and race/ethnicity. Model II adjusted for age, sex, race/ethnicity, smoking, drinking, education levels, hypertension, diabetes, dyslipidemia, physical inactivity, and obesity.

*OR* odds ratio, *CI* confidence interval, *HEI* healthy eating index, *Q1* 1st quartile, *Q2* 2nd quartile, *Q3* 3rd quartile, *Q4* 4th quartile.

***P value < 0.001, **P value < 0.01.

### LASSO regression and nomogram model

**Figure 4:**
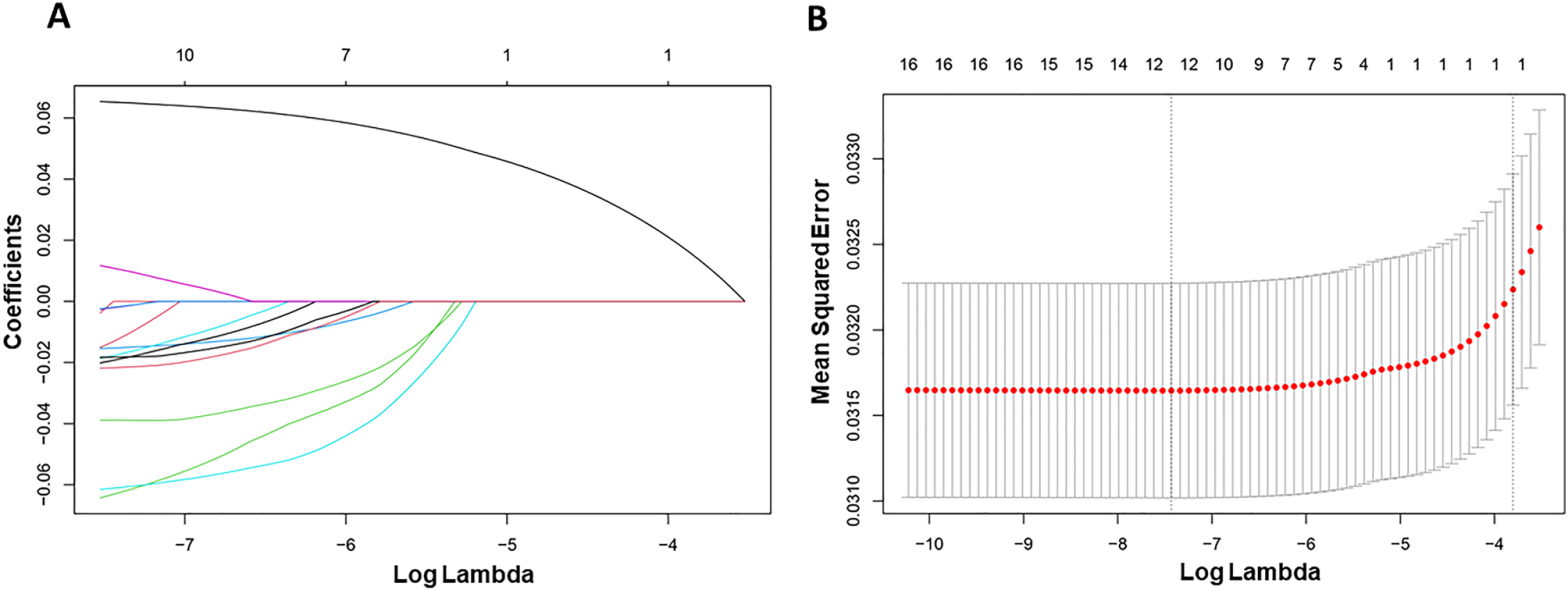
LASSO regression analysis to screen out key dietary factors most related to stroke. (**A**) Plot for LASSO regression coefficients. (**B**) Cross-validation plot.

**Figure 5:**
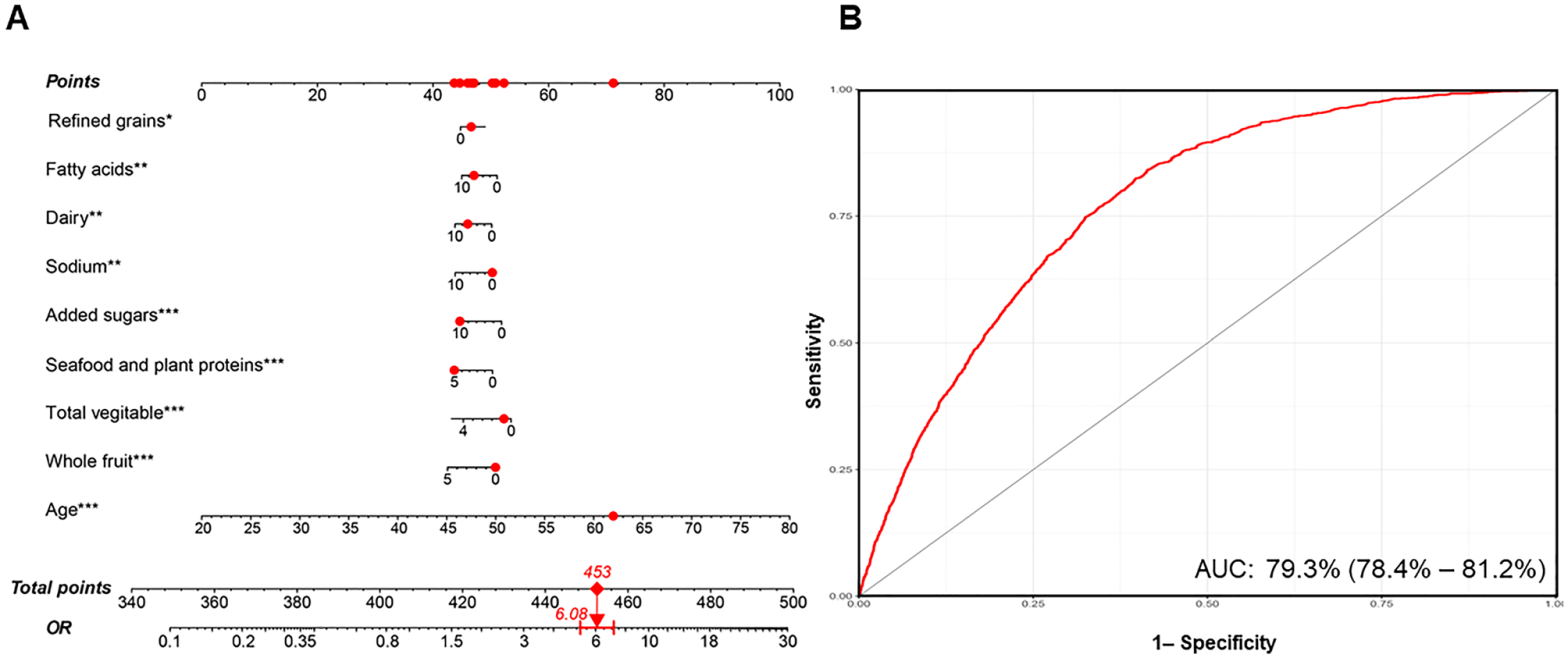
Nomogram model established for predicting the risk of stroke and its ROC curve. (**A**) Nomogram model based on the key dietary factors screened out by LASSO regression, and the red points show an example: for 62-year-old male participants with stroke, the probability of stroke increased by 6.08-fold. (**B**) ROC curve for evaluating the diagnostic power of the nomogram model in this study.

LASSO regression was utilized to identify the dietary factors most strongly associated with stroke (Fig. 4A, B). Eight variables (refined grains, fatty acids, dairy, sodium, added sugars, seafood and plant proteins, total vegetable, whole fruit, and age) were included in the final model due to their statistical significance (Fig. 5A). The ROC results showed that our nomogram model had favorable discriminatory power, with an AUC of 79.3% (95% CI 78.4–81.2%) (Fig. 5B).

### Sensitive analysis

We also carried out a sensitive analysis in which unweighted logistic analysis on the association between HEI and the risk of stroke was carried out. We found that the results of the sensitivity analysis were consistent with the results of the main analysis. In the fully adjusted logistic regression model, HEI was negatively associated with stroke; participants in higher quartiles determined by HEI were less likely to have a stroke (Q2: OR 0.89, 95% CI 0.73–1.06; Q3: OR 0.78, 95% CI 0.67–0.92; Q4: OR 0.66, 95% CI 0.56–0.72) (Table 4). Thus, the results of the sensitivity analysis indicated that the findings of the current study are robust and dependable, showing a negative correlation between an increase in HEI and the incidence of stroke.

**Table 4:** Unweighted logistic regression analysis on the association between HEI and stroke in sensitive analysis.

	Non-adjusted model		Model I		Model II	
	OR [95% CI]	*P* value	OR [95% CI]	*P* value	OR [95% CI]	*P* value
Q1	Reference	–	Reference	–	Reference	–
Q2	0.95 (0.82, 1.09)	0.46	0.83 (0.72, 0.95)	0.01*	0.89 (0.73, 1.06)	0.06
Q3	0.89 (0.77, 1.02)	0.10	0.68 (0.59, 0.79)	< 0.001***	0.78 (0.67, 0.92)	< 0.001***
Q4	0.79 (0.68, 0.91)	0.001**	0.52 (0.45, 0.60)	< 0.001***	0.66 (0.56, 0.72)	< 0.001***

Data are presented as OR [95% CI]. Model I adjusted for age, sex, and race/ethnicity. Model II adjusted for age, sex, race/ethnicity, smoking, drinking, education levels, hypertension, diabetes, dyslipidemia, physical inactivity, and obesity.

*OR* odds ratio, *CI* confidence interval, *HEI* healthy eating index, *Q1* 1st quartile, *Q2* 2nd quartile, *Q3* 3rd quartile, *Q4* 4th quartile.

***P value < 0.001, **P value < 0.01, *P value < 0.05.

## Discussion

The study aimed to explore the association between the healthy eating index (HEI) and stroke prevalence in a diverse U.S. population. Using cross-sectional data from the National Health and Nutrition Examination Survey (NHANES), dietary information was collected, and HEI scores were computed. Participants with a history of stroke had lower HEI scores compared to those without stroke, indicating a robust association even after adjusting for confounding variables. RCS analysis indicated a nonlinear negative correlation between HEI and stroke risk. Subgroup analysis revealed a gender-based disparity, suggesting potential greater benefits for women from dietary quality improvements. The nomogram model, based on LASSO regression-identified dietary factors, demonstrated favorable discriminatory power (AUC: 79.3%, 95% CI 78.4–81.2%). Higher HEI scores were inversely related to stroke risk, emphasizing the importance of improving dietary quality for enhanced stroke prevention, particularly for women.

The healthy eating index (HEI) is a tool that measures the quality of an individual's diet based on the recommendations of the Dietary Guidelines for Americans. The HEI was first developed by the United States Department of Agriculture (USDA) in 1995 and has since been updated periodically to reflect new scientific knowledge and changes in dietary guidelines^20^. The HEI is composed of 12 components, each of which reflects a specific aspect of a healthy diet. These components include the intake of fruits, vegetables, whole grains, dairy products, protein foods, added sugars, saturated fats, and sodium^21^. Additionally, the HEI includes two components that reflect the overall diet quality, namely, variety and moderation. Each component is scored from 0 to 10, with a maximum total score of 100 indicating a diet that meets all the recommendations of the Dietary Guidelines for Americans^22^. The HEI has been used in various settings, including clinical practice, public health research, and dietary surveillance^23–25^. In clinical practice, the HEI can be used to assess the quality of an individual’s diet and provide guidance on dietary modifications to improve diet quality. In research, the HEI has been used to investigate the relationship between diet quality and various health outcomes, such as cardiovascular disease, cancer, and diabetes^26,27^. The HEI has also been used in dietary surveillance to monitor the diet quality of the population over time and to identify subgroups that may be at risk for poor diet quality. It has been shown to be a strong predictor of overall diet quality, nutrient adequacy, and health outcomes. A higher HEI score has been associated with a lower risk of chronic diseases, such as coronary artery disease, and overall mortality^23^. One of the strengths of the HEI is its flexibility in application. It can be used to assess the diet quality of individuals, households, or populations, and can be adapted to reflect cultural and regional dietary patterns^23^. The HEI has also been used to evaluate the effectiveness of dietary interventions, such as nutrition education programs and food assistance programs^28,29^. However, the application of HEI in the field of predicting stroke risk and prevention and treatment of stroke is still limited.

Numerous studies have investigated the effects of dietary patterns and dietary components on the risk of stroke. These studies have provided valuable insights into the potential impact of dietary modifications on stroke prevention. Several large prospective cohort studies have consistently reported that adherence to a healthy dietary pattern, such as the Mediterranean diet and DASH diet is associated with a reduced risk of stroke^30,31^. In addition to overall dietary patterns, specific dietary components have also been investigated for their impact on stroke risk. A meta-analysis by Dan et al*.* reported that higher consumption of fruits and vegetables was associated with a reduced risk of stroke, with a 32% (0.68 [0.56–0.82]) and 11% (0.89 [0.81–0.98]) for every 200 g per day increment in fruits consumption (p for nonlinearity = 0.77) and vegetables consumption (p for nonlinearity = 0.62), respectively^32^. A separate meta-analysis by Angela et al*.* reported that high consumption of whole grains was associated with a lower risk of stroke, with an 8% reduction in risk observed for every 3 servings per day increase in whole grain intake^33^. Several studies have also investigated the impact of dietary factors on specific subtypes of stroke. For example, a study by Diane et al*.* reported that a higher intake of dietary fiber was associated with a reduced risk of ischemic stroke, but not hemorrhagic stroke^34^. Similarly, a study by Larsson et al*.* reported that a high intake of fish was associated with a lower risk of ischemic stroke, but not hemorrhagic stroke^35^. While these studies provide valuable insights into the potential impact of dietary modifications on stroke prevention, it is important to note that the results are not always consistent across studies. For example, a meta-analysis by Wang et al*.* reported that high consumption of red and processed meat was associated with an increased risk of stroke, but several other studies have not found a significant association^36^. Overall, the evidence suggests that dietary modifications can play an important role in stroke prevention. Adherence to a healthy dietary pattern, such as the Mediterranean diet or DASH diet, as well as higher consumption of fruits, vegetables, whole grains, and fish, may be particularly beneficial in reducing the risk of stroke. However, further research is needed to better understand the mechanisms underlying these associations and to identify the most effective dietary modifications for stroke prevention. Besides, it is significantly needed to establish a measurement to evaluate the overall dietary quality and explore its association with various diseases. In the present study, we found that HEI, as an overall index evaluating dietary quality, was closely associated with the risk of stroke and can be used as a prediction parameter with favor diagnostic value.

Moreover, we also noticed that there was a significant sex difference in the specific impact of dietary quality on the risk of stroke. There is already evidence to suggest that there are gender differences in the impact of dietary quality on the occurrence of various diseases. In a recent large-scale prospective study, researchers examined the impact of diet quality on cardiovascular diseases. The study involved 155,724 participants from 21 different countries who were tracked for approximately 10 years. The study’s primary objective was to measure major cardiovascular events such as cardiovascular death, myocardial infarction, stroke, and heart failure. The results indicated that low diet quality had a stronger association with cardiovascular diseases in female participants than in male participants. The hazard ratio for females was 1.17 (95% CI 1.08–1.26), while for males, it was 1.07 (0.99–1.15)^37^. There are several potential explanations for the gender differences in the impact of dietary quality on stroke risk. Firstly, women may have a higher susceptibility to the negative effects of a poor diet due to differences in physiology and hormonal factors. For example, estrogen is known to have a protective effect on the cardiovascular system, and a decline in estrogen levels during menopause may increase the risk of CVD and stroke in men^10^. Additionally, men may have a lower capacity to metabolize and eliminate dietary fats, leading to higher levels of circulating lipids and an increased risk of atherosclerosis^11^. Secondly, there may be differences in dietary patterns between men and women that contribute to the observed gender differences in stroke risk. For example, men may be more likely to consume a diet high in refined carbohydrates, which are associated with a higher risk of CVD and stroke^38^. On the other hand, men may be more likely to consume a diet high in saturated fats, which are also linked to an increased risk of CVD and stroke^39^. Several studies have investigated the relationship between dietary quality and stroke risk, specifically in women. For instance, Akbaraly et al*.* reported that adherence to a healthy diet was associated with a lower risk of stroke in women, but not in men^40^. These findings suggest that improving dietary quality may be a more effective strategy for reducing stroke risk in women. In addition to reducing stroke risk, a healthy diet may also have other beneficial effects on women’s health. For example, a diet rich in fruits and vegetables is associated with a lower risk of breast cancer, and a diet high in omega-3 fatty acids may reduce the risk of depression and improve cognitive function^41,42^.

Studying the HEI is important because it allows researchers and healthcare professionals to identify specific dietary patterns and components that are associated with a lower risk of stroke. This information can be used to develop targeted interventions to improve dietary quality and reduce the incidence of stroke. Diagnostic prediction models are mathematical tools that use a combination of clinical and non-clinical variables to predict the probability of a specific outcome, such as stroke. These models can be used to identify individuals who are at high risk of stroke and implement targeted interventions to reduce their risk. The HEI can be incorporated into diagnostic prediction models for stroke because it provides information on an individual's dietary quality, which is a modifiable risk factor for stroke.

In the present study, we also established a diagnostic model, which included refined grains, fatty acids, dairy, sodium, added sugars, seafood and plant proteins, total vegetables, and whole fruit. The ROC results showed that our nomogram model had favorable discriminatory power, with an AUC of 79.3% (95% CI 78.4–81.2%). However, we believe a more precise model including more dietary components is needed. In the future, the development of diagnostic prediction models based on dietary habits for stroke that incorporate the HEI has important implications for public health. By identifying individuals who are at high risk of stroke, healthcare professionals can implement targeted interventions to reduce their risk, including dietary interventions to improve dietary quality. In addition, the use of the HEI in diagnostic prediction models for stroke can inform public health policies and guidelines.

There are several implications and advantages stemming from this study. Firstly, due to the large population included in this study and the fact that all statistical processes were weighted and followed NHANES guidelines, reliable conclusions can be drawn. As a result, the conclusions drawn from this study may be applicable to the 201 million residents of the United States. Secondly, we utilized LASSO regression analysis to identify key dietary factors that are closely related to stroke, and we established a nomogram model with a strong discriminatory ability. Despite these benefits, it is important to note some limitations of this study. For instance, this study is cross-sectional in nature, and therefore, a causal relationship cannot be established. More prospective studies are needed, and the application of HEI in the stroke field may be limited. Additionally, subjective bias may exist due to self-reported dietary information and covariates from the NHANES database. Furthermore, daily diets can vary widely, and a 24-h recall of dietary information may be insufficient. Significant ethnic differences exist in terms of diet, physical activity, genetic variations, lipid metabolism, and susceptibility to cardiovascular disease. As a result, it is necessary to investigate whether the conclusions of this study based on US participants can be extended to other populations in future research. Moreover, another limitation is important to note that stroke survivors from NHANES, may not be fully representative of the entire stroke survivor population. The NHANES dataset does not include individuals who are hospitalized or residing in care facilities. Besides, While our study utilized an average of 2 recalls, the potential measurement error intrinsic to repeated 24-h dietary recalls and recall bias may exist.

## Conclusions

A retrospective analysis was conducted on 43,978 participants from the NHANES survey, which revealed a sex difference in the association between the HEI and the risk of stroke. Using LASSO regression, key dietary factors that were mostly linked to stroke were identified. A nomogram model was established based on these key factors, which exhibited favorable diagnostic power in predicting the risk of stroke. The study highlights the importance of paying more attention to HEI and improving dietary quality for preventing and treating stroke. However, further prospective studies are still needed in this regard.

### Supplementary Information

Supplementary Table S1.

Supplementary Table S2.

## Data Availability

Publicly available datasets were analyzed in this study. All the raw data used in this study are derived from the public NHANES data portal (https://wwwn.cdc.gov/nchs/nhanes/analyticguidelines.aspx).
