# Supplementary material for: Healthy eating index-2015 and its association with the prevalence of stroke among US adults
Source: Sci Rep. 2024 Feb 12;14:3516. doi: 10.1038/s41598-024-54087-9 (PMC10861484; doi:10.1038/s41598-024-54087-9)
Supplement: Supplementary file 1 — Supplementary Table S1. [file 41598_2024_54087_MOESM1_ESM.docx]

**Table S1. Baseline Characteristics Among Different HEI Quartiles.**

| Variables | HEI-Q1 | HEI-Q2 | HEI-Q3 | HEI-Q4 | *P* value |
| --- | --- | --- | --- | --- | --- |
| Age, years | 42.22 [41.85, 42.58] | 44.87 [44.45, 45.29] | 46.66 [46.16, 47.15] | 49.78 [49.25, 50.32] | < 0.001*** |
| Sex-male, *n* (%) | 52.26 [51.07, 53.46] | 51.00 [49.83, 52.17] | 49.46 [48.34, 50.58] | 44.44 [43.38, 45.50] | < 0.001*** |
| Race, *n* (%) |  |  |  |  |  |
| Non-Hispanic White | 69.21 [66.90, 71.53] | 67.39 [65.01, 69.77] | 67.20 [64.96, 69.45] | 69.12 [67.10, 71.14] | < 0.001*** |
| Non-Hispanic Black | 12.57 [11.22, 13.92] | 12.53 [11.15, 13.91] | 10.83 [9.63, 12.02] | 8.73 [7.76, 9.70] |  |
| Mexican American | 7.69 [6.56, 8.83] | 8.60 [7.42, 9.77] | 9.09 [7.87, 10.32] | 7.56 [6.52, 8.60] |  |
| Other Hispanic | 5.22 [4.26, 6.17] | 5.42 [4.40, 6.44] | 5.89 [4.95, 6.84] | 6.20 [5.19, 7.22] |  |
| Other | 5.31 [4.66, 5.96] | 6.06 [5.40, 6.72] | 6.98 [6.21, 7.75] | 8.39 [7.53, 9.24] |  |
| Smoking, *n* (%) | 31.11 [29.83, 32.39] | 25.80 [24.59, 27.01] | 19.90 [18.85, 20.96] | 11.71 [10.85, 12.57] | < 0.001*** |
| Drinking, *n* (%) | 89.76 [88.94, 90.59] | 90.72 [89.60, 91.83] | 88.60 [87.43, 89.77] | 87.64 [86.41, 88.87] | < 0.001*** |
| Education level, *n* (%) |  |  |  |  |  |
| Below high school | 5.00 [4.45, 5.54] | 5.61 [5.01, 6.21] | 5.92 [5.36, 6.49] | 5.19 [4.61, 5.76] | < 0.001*** |
| High school | 43.99 [42.29, 45.68] | 37.91 [36.39, 39.42] | 33.14 [31.64, 34.64] | 25.44 [24.06, 26.82] |  |
| Above high school | 51.02 [49.27, 52.77] | 56.48 [54.79, 58.18] | 60.94 [59.33, 62.54] | 69.37 [67.80, 70.94] |  |
| SBP, mmHg | 121.25 [120.82, 121.67] | 121.64 [121.19, 122.09] | 121.80 [121.32, 122.27] | 121.78 [121.25, 122.31] | 0.18 |
| DBP, mmHg | 72.05 [71.65, 72.46] | 71.83 [71.49, 72.18] | 71.67 [71.30, 72.04] | 71.03 [70.65, 71.41] | < 0.001*** |
| Diabetes, *n* (%) | 11.25 [10.49, 12.02] | 12.29 [11.56, 13.03] | 12.59 [11.80, 13.37] | 13.27 [12.46, 14.07] | 0.001** |
| eGFR, ml/min/1.73m^2^ | 97.81 [97.19, 98.43] | 96.21 [95.58, 96.84] | 94.57 [93.86, 95.27] | 92.17 [91.48, 92.85] | < 0.001*** |
| RBC, ×10^9^/L | 4.78 [4.76, 4.79] | 4.74 [4.73, 4.76] | 4.72 [4.71, 4.74] | 4.67 [4.65, 4.68] | < 0.001*** |
| WBC, ×10^9^/L | 7.60 [7.54, 7.66] | 7.37 [7.31, 7.43] | 7.21 [7.15, 7.27] | 6.82 [6.76, 6.88] | < 0.001*** |
| NE, ×10^9^/L | 4.52 [4.48, 4.57] | 4.39 [4.34, 4.43] | 4.27 [4.22, 4.31] | 4.00 [3.96, 4.05] | < 0.001*** |
| Monocyte, ×10^9^/L | 0.58 [0.57, 0.58] | 0.56 [0.56, 0.57] | 0.56 [0.55, 0.57] | 0.54 [0.53, 0.54] | < 0.001*** |
| LY, ×10^9^/L | 2.24 [2.21, 2.27] | 2.17 [2.15, 2.19] | 2.13 [2.11, 2.16] | 2.04 [2.02, 2.07] | < 0.001*** |
| PLT, ×10^6^/L | 259.83 [258.06, 261.61] | 258.27 [256.17, 260.37] | 254.20 [252.36, 256.05] | 246.93 [245.10, 248.77] | < 0.001*** |
| Hemoglobin, g/L | 14.45 [14.39, 14.50] | 14.39 [14.34, 14.44] | 14.38 [14.32, 14.44] | 14.25 [14.20, 14.29] | < 0.001*** |

Continuous variables are presented as the mean [95% CI], category variables are presented as the proportion [95% CI]. CI, confidence interval; SBP, systolic blood pressure; DBP, diastolic blood pressure; eGFR, estimated glomerular filtration rate; BMI, body mass index; WC, waist circumference; RBC, red blood cells; WBC, white blood cells; NE, neutrophils; LY, lymphocytes; PLT, platelets. *** *P* value<0.001
