# Supplementary material for: Healthy eating index-2015 and its association with the prevalence of stroke among US adults
Source: Sci Rep. 2024 Feb 12;14:3516. doi: 10.1038/s41598-024-54087-9 (PMC10861484; doi:10.1038/s41598-024-54087-9)
Supplement: Supplementary file 2 — Supplementary Table S2. [file 41598_2024_54087_MOESM2_ESM.docx]

**Table S2. Comparison of Cardiometabolic Measurements Among Different HEI Quartiles.**

| Variables | HEI-Q1 | HEI-Q2 | HEI-Q3 | HEI-Q4 | *P* value |
| --- | --- | --- | --- | --- | --- |
| FBG, mmol/L | 5.83 [5.78, 5.88] | 5.87 [5.81, 5.93] | 5.82 [5.77, 5.88] | 5.82 [5.76, 5.89] | 0.62 |
| FBI, pmol/L | 85.00 [82.05, 87.94] | 80.95 [77.39, 84.52] | 75.25 [72.45, 78.05] | 68.29 [65.85, 70.73] | < 0.001*** |
| HOMA-IR | 3.97 [3.79, 4.14] | 3.81 [3.59, 4.03] | 3.49 [3.34, 3.64] | 3.24 [3.07, 3.40] | < 0.001*** |
| HbA1c, % | 5.56 [5.54, 5.58] | 5.57 [5.55, 5.60] | 5.57 [5.55, 5.60] | 5.57 [5.55, 5.60] | 0.74 |
| TG, mmol/L | 1.55 [1.50, 1.59] | 1.52 [1.48, 1.56] | 1.53 [1.47, 1.59] | 1.40 [1.37, 1.44] | < 0.001*** |
| TC, mmol/L | 5.04 [5.01, 5.06] | 5.09 [5.06, 5.12] | 5.12 [5.09, 5.15] | 5.09 [5.06, 5.12] | < 0.001*** |
| HDL-C, mmol/L | 1.29 [1.27, 1.30] | 1.34 [1.33, 1.36] | 1.38 [1.37, 1.40] | 1.46 [1.44, 1.47] | < 0.001*** |
| LDL-C, mmol/L | 3.02 [2.98, 3.05] | 3.02 [2.99, 3.06] | 3.01 [2.97, 3.04] | 2.98 [2.94, 3.01] | 0.25 |
| CRP, mg/dl | 0.45 [0.43, 0.47] | 0.43 [0.40, 0.46] | 0.38 [0.36, 0.40] | 0.35 [0.33, 0.37] | < 0.001*** |

Data are presented as the mean [95% CI]. FBG, fasting blood glucose; FBI, fasting blood insulin; HOMA-IR, homeostasis model assessment to evaluate insulin resistance; HbA1c, glycated hemoglobin; TG, triglycerides; TC, total cholesterol; HDL-C, high-density lipoprotein cholesterol; LDL-C, low-density lipoprotein cholesterol; CRP, C-reactive protein.
